# Imaging the socially-anxious brain: recent advances and future prospects

**DOI:** 10.12688/f1000research.21214.1

**Published:** 2020-04-02

**Authors:** Janna Marie Bas-Hoogendam, P. Michiel Westenberg

**Affiliations:** 1 Developmental and Educational Psychology, Institute of Psychology, Leiden University, Wassenaarseweg 52, 2333 AK Leiden, The Netherlands; 2 Leiden Institute for Brain and Cognition, c/o LUMC, postzone C2-S, P.O.Box 9600, 2300 RC Leiden, The Netherlands; 3 Department of Psychiatry, Leiden University Medical Center, Albinusdreef 2, 2333 ZA Leiden, The Netherlands

**Keywords:** social anxiety, MRI, biomarkers, endophenotypes

## Abstract

Social anxiety disorder (SAD) is serious psychiatric condition with a genetic background. Insight into the neurobiological alterations underlying the disorder is essential to develop effective interventions that could relieve SAD-related suffering. In this expert review, we consider recent neuroimaging work on SAD. First, we focus on new results from magnetic resonance imaging studies dedicated to outlining biomarkers of SAD, including encouraging findings with respect to structural and functional brain alterations associated with the disorder. Furthermore, we highlight innovative studies in the field of neuroprediction and studies that established the effects of treatment on brain characteristics. Next, we describe novel work aimed to delineate endophenotypes of SAD, providing insight into the genetic susceptibility to develop the disorder. Finally, we outline outstanding questions and point out directions for future research.

## Introduction

Social anxiety disorder (SAD) is a serious psychiatric condition with a genetic background and typically evolves during late childhood and early adolescence ^1– 5^. Patients are afraid of a negative evaluation by others and avoid social situations as much as possible, leading to significant adverse effects on important areas of functioning ^6– 9^. SAD is characterized by a chronic course, has severe consequences for patients ^10– 14^ and high costs for society ^15^, and is often suboptimally treated ^16, 17^. In order to develop effective interventions, which could relieve individual suffering and reduce the serious societal consequences of SAD, insight into the neurobiological alterations underlying the disorder is essential ^18^. We are pleased to note that SAD is increasingly considered an interesting topic worthy of investigation ^19^. In this review, we highlight recent advances with respect to neuroimaging work on SAD while focusing on data from magnetic resonance imaging (MRI) studies. First, we review work on biomarkers for SAD, being any measurable indicator of disease; second, we summarize recent work on profiling SAD endophenotypes, which are heritable and measurable characteristics associated with a certain disorder ^20^. It is essential to distinguish biomarkers from endophenotypes, as they provide different types of information. For example, biomarker research is valuable for identifying treatment targets, whereas endophenotypes may be important for disentangling genetic underpinnings and identifying genetic mediators. Subsequently, we outline several outstanding questions and provide suggestions for future studies.

## Biomarker research on social anxiety disorder

As recently outlined by Etkin ^21^, neuroimaging research in psychiatry often uses a case-control design, in which a selected group of patients, based mostly on meeting the *Diagnostic and Statistical Manual of Mental Disorders* (DSM) criteria for a specific disorder, is compared with a sample of healthy control participants. Such a design is especially suitable to identify biomarkers, as these are features associated with a particular condition. In 2014, Brühl *et al*. published an extensive overview of the neuroimaging biomarker literature on SAD ^22^. They summarized findings with respect to the structure and function of the brain in patients with SAD, reviewed data on brain connectivity in SAD, and performed a meta-analysis of functional MRI (fMRI) studies regarding brain responsiveness in SAD. Their overview resulted in a neurofunctional model of the socially anxious brain, characterized by hyperactivation of the fear circuit—consisting of the amygdala, insula, prefrontal cortex (PFC) and anterior cingulate cortex (ACC)—and hyper-responsive parietal and occipital regions, in reaction to SAD-relevant stimuli. Furthermore, Brühl *et al*. described SAD-related hypoconnectivity of these parieto-occipital brain areas, which are located in the medial part of the brain and are part of the default mode network and dorsal attention network; thereby, the authors suggest that these changes in network structure are reflective of the increased emotional arousal and enhanced focus on potentially threatening stimuli that characterize patients with SAD ^22^.

Since the publication of that review ^22^, new findings have added to our knowledge of the neurobiological basis of SAD. Several recent structural MRI studies have pointed to a role of the striatum in SAD and in related constructs such as “intolerance of uncertainty” ^23^; these studies, which concern (respectively) an international mega-analysis in the largest MRI sample of 174 patients with SAD and 213 healthy controls to date ^24^, a study in women with a varying range of social anxiety levels ^25^, and a correlational study in a sample of 61 healthy volunteers ^26, 27^, all implied increased striatal volume in (social) anxiety. Interestingly, a treatment study on SAD revealed that 8-weeks of paroxetine treatment led to significant reductions in gray matter in the bilateral caudate and putamen, both part of the striatum ^28^. However, another study reported decreased gray matter in the putamen in SAD ^29^. Furthermore, the increase in striatum volume was not replicated by a recent meta-analysis on brain structure in SAD ^30^; see also the commentary belonging to this work ^31^. These inconsistent findings underscore the need for replication studies on large datasets (for example, by pooling data from different research centers, which use preferably similar protocols for data acquisition and analysis) (compare the recommendation by 21). In recent years, interest in data sharing has grown, and initiatives such as Enhancing Neuro Imaging by Meta-Analysis (ENIGMA) ^32^ have increasingly received attention (see a summary of a decade of ENIGMA studies in 33); analyses on brain structure in SAD are currently being performed within the ENIGMA-Anxiety Working Group ^34^.

In addition to these structural MRI studies, recent fMRI studies explored the relationship between social anxiety and brain responses, aiming to identify functional biomarkers. Several research directions deserve to be highlighted. An intriguing line of research focuses on amygdala activation in social anxiety. This is not surprising, as the amygdala, a small structure located deep in the brain, plays a critical role in detecting and evaluating environmental cues that might be reflective of potential threat ^35, 36^, and amygdala hyperreactivity in SAD has been frequently reported ^37^. Interestingly, recent work aims to determine in more detail which functional alterations in amygdala responses are associated with social anxiety. We will mention several lines of research.

First of all, an influential article published around 20 years ago provided evidence that amygdala activation, in response to stimuli presented multiple times without a meaningful consequence, decreased over time (that is, higher amygdala activation was present for novel faces when compared with already presented faces, a process called habituation) ^38^; subsequently, a landmark article indicated that adult participants who were characterized at age 2 as “behavioral inhibited” (a temperamental trait associated with an increased risk for developing SAD later in life ^39– 42^) showed increased amygdala responses to novel faces ^43^, providing an initial explanation for their anxious feelings in social situations. Building upon these findings, work from 2011 showed that individuals with an extreme inhibited temperament were characterized by a sustained increase in amygdala activation to faces ^44^, and a follow-up article confirmed that the amygdala response failed to habituate in participants with an inhibited temperament ^45^. In 2016, Avery and Blackford demonstrated that habituation rate differed across the continuum of social fearfulness, and slower rates of habituation in participants were associated with higher levels of social fearfulness ^46^; furthermore, these differences were not limited to the amygdala but were present in multiple regions of the social brain, and connectivity analyses revealed that slower habituation was accompanied by increased connectivity between the amygdala and visual brain areas ^46, 47^. Although these findings need to be replicated in patients with SAD, this series of experiments increases our insight into the failed habituation response in socially anxious participants and provides a neurobiological basis for their experience of feeling uncomfortable in social settings.

Other lines of amygdala research are devoted to determining the specificity of SAD-related amygdala hyperactivation ^48^, the time course of amygdala activation ^49, 50^, and the specific roles of amygdala sub-regions during aversive processing ^51^. In addition, studies on task-related connectivity of the amygdala—for example, during emotion discrimination ^52^, related to performing an affective counting Stroop task with emotional faces ^53^, and during the perception of fearful faces ^54^ or disorder-related complex visual scenes ^55^—revealed that SAD is not characterized just by aberrant local amygdala activation; instead, the whole functional amygdala network displays alterations, and the direction of these changes depends on the specific task on hand.

Furthermore, the role of the bed nucleus of stria terminalis (BNST), as part of the extended amygdala network, has increasingly received attention in research on threat processing and in studies on (social) anxiety ^56– 66^ . The role of the BNST in threat and anxiety (in comparison with the function of the amygdala) is still a topic of debate; whereas some researchers attributed a specific and unique functional role to the BNST when compared with the amygdala ^63, 67^, others indicate that the BNST and amygdala have similar functional profiles ^68^. When we specifically consider BNST research in SAD, evidence for both viewpoints is provided. On the one hand, Blackford *et al*. (2019) demonstrated that social anxiety was associated with a BNST versus amygdala difference in response to unpredictable images ^65^. On the other hand, a recent study on the temporal profile of amygdala and BNST activation during the anticipation of temporally unpredictable aversive cues revealed similar activation patterns in the central amygdala and BNST: the investigators reported increased phasic activation in both the amygdala and BNST in patients with SAD compared with healthy controls, possibly reflective of hypervigilance in the SAD group; no group differences were present when sustained brain activation (in both amygdala and BNST) was considered ^69^. We argue that, in line with the comments of Gungor and Pare included in the article by Shackman and Fox ^68^, future research on fear and anxiety should not ignore the BNST but needs to acknowledge this structure as part of the integral anxiety network.

Next, we want to acknowledge recent studies on alterations in functional connectivity (FC) of the brain during rest ^70– 75^; cf. the review by MacNamara *et al*. ^76^. It should be mentioned that these studies often differ in their analytical approaches and investigate various networks of interest. A meta-analysis of their findings is beyond the scope of this expert review, but most studies report changes in the default mode network, a network involved in social referencing and the cognitive ability to understand other people’s mental state (theory of mind) ^77^. Interestingly, a study using multivariate pattern analysis demonstrated that patients with SAD could be reliably classified (versus healthy controls) on the basis of FC measures ^78^. Furthermore, recent studies using graph theory models provided insight into the topological organization of functional networks in SAD ^79– 82^.

A new line of biomarker research in SAD focuses on determining reliable biomarkers for treatment choice. (For a review on neuroprediction in anxiety disorders, we recommend ^83^; a review dedicated to SAD was recently provided by Klumpp and Fitzgerald ^84^.) For example, Frick *et al*. ^85^ investigated a sample of 48 patients with SAD and acquired fMRI scans before the start of a 9-week treatment with internet-based cognitive behavioral therapy (CBT), CBT plus the serotonin reuptake inhibitor escitalopram, or a placebo. The investigators explored how pre-treatment brain responses related to treatment outcome, and they demonstrated that pre-treatment brain activation in the dorsal ACC predicted the response to CBT ^85, 86^. Other recent examples of work on neuroprediction in SAD report that the outcome of CBT could be predicted from pre-treatment activation in the dorsolateral PFC ^87^, frontoparietal regions (including the dorsal ACC and insula) ^88^, and the rostral ACC ^89^. In addition, several research groups explored the use of brain connectomics as predictive biomarkers for treatment response to CBT. One group used the amygdala as a seed region and demonstrated that resting-state connectivity and integrity of a specific white matter tract (the right inferior longitudinal fasciculus) predicted clinical improvement in patients with SAD ^90^; another group recently showed that stronger inverse FC between the amygdala and the ventrolateral PFC, as measured during an implicit emotion regulation task, was related to better treatment response ^91^. Furthermore, activation in the ventromedial PFC during early extinction learning predicted the reduction of public speaking anxiety and social anxiety symptoms after exposure ^92^. Although these studies show the potential clinical relevance of neuroimaging in deciding on appropriate treatment options for patients with SAD, the results should be considered preliminary given the small sample sizes and heterogeneous findings (cf. 83, 84).

Related to research on neuroprediction are recent studies focusing on the effect of treatment on the brain. Using a longitudinal design and multiple MRI techniques, Steiger *et al*. demonstrated changes in several structural brain characteristics following group CBT ^93^. Another multimodal longitudinal study reported decreases in amygdala volume and amygdala activation levels after CBT and showed that the reduction in amygdala volume mediated the relationship between the diminished amygdala response and clinical improvement ^94^. Notably, in a one-year follow-up study on the same participants, the investigators still found reduced amygdala volume in CBT responders; this finding suggests that effective psychological interventions can induce long-lasting changes in human brain structure ^95^. However, given the small sample size (n = 13 patients with SAD) in the latter study, more longitudinal studies are essential to further substantiate this finding and to explore the long-term effects of treatment on brain function. Other studies demonstrated effects of treatment on FC between the amygdala and ventromedial PFC ^96^ and on network parameters of the precuneus ^97^.

Importantly, the majority of studies summarized above were performed in adult patients with SAD. However, given the early onset of the disorder, typically during late childhood and early adolescence ^98^, neuroimaging studies in socially anxious youth can provide valuable information about the neurobiology of the disorder, as the results of these studies are less likely to be confounded by the experience of recurrent SAD episodes. On the other hand, most neuroimaging studies on adolescents include comorbid anxiety disorders (for example, interesting work on attentional processing of social threat and responses to social evaluation ^99– 104^; a review of neuroimaging in pediatric anxiety is available here ^105^), which makes it difficult to establish specific SAD-related neurobiological alterations (see the work of McElroy *et al*. demonstrating strong associations between symptoms of depression and anxiety in childhood and early to mid-adolescence ^106^). Nevertheless, several studies provided insight into the neural substrates of SAD across developmental phases. Blair *et al*. (2011) ^107^, for example, revealed increased brain responses in the amygdala and rostral ACC in both adult and adolescent participants with SAD, suggesting that the neurobiological characteristics of adults with SAD are not the result of adaptive responses or developmental changes over time; rather, these alterations are stable characteristics related to the disorder. On the other hand, work of Britton *et al*. (2013) in anxious adolescents and adults distinguished shared and age-specific neurobiological correlates of fear conditioning ^108^, and Jarcho *et al*. reported on heightened striatal activity in adolescents with SAD but not in socially anxious adults ^109^. In addition, there is work on adolescents who are at risk for developing SAD, based on the fact that they are characterized as “behavioral inhibited”; such studies demonstrated, for example, increased amygdala response ^110^ and altered striatal activation ^111, 112^ in children and adolescents temperamentally at risk for anxiety. (An extensive review of literature on this topic is provided elsewhere ^113^; for a more complete overview of neuroimaging work in adolescent SAD, we recommend ^1, 114^.) Longitudinal studies (cf. 115), preferably following children into adulthood, are essential to shed light on this important topic ^116^.

## Endophenotype research on social anxiety disorder

In addition to highlighting these biomarker studies, we want to mention recent work on SAD endophenotypes. Endophenotypes are measurable and heritable characteristics on the pathway from genotype to phenotype; as defined in literature on this topic ^117, 118^, endophenotypes are supposed to be (1) associated with a particular disorder, (2) stable trait characteristics, and (3) heritable. Furthermore, endophenotypes should co-segregate with the disorder of interest within families, and non-affected family members show altered levels of the endophenotype in comparison with the general population (fourth criterion). Thereby, endophenotypes are reflective of the genetic vulnerability to develop psychopathology and this important characteristic distinguishes endophenotypes from biomarkers. Biomarkers do not necessarily have a genetic basis; endophenotypes, on the other hand, are by definition heritable and supposed to be reflective of genetically based disease mechanisms ^20^. So, as stated by Lenzenweger, “all endophenotypes are biomarkers, but not all biomarkers are endophenotypes” ^20^. Given their genetic background, endophenotypes can be used to unravel the genetic vulnerability to psychopathology. In addition, endophenotypes provide insight into the pathways leading to complex psychiatric disorders ^119, 120^. Furthermore, endophenotypes can increase our understanding of the transdiagnostic characteristics of mental disorders ^120, 121^.

In the past decade, the endophenotype approach has been applied to psychiatric disorders such as depression ^122, 123^, obsessive–compulsive disorder ^124– 126^, and schizophrenia ^127– 130^, revealing alterations in brain structure and function in patients and their unaffected relatives. Thereby, these studies provide initial insight into the genetic vulnerability to these disorders, as they show that the changes are not just a manifestation of the disease state (as the alterations were present in unaffected family members as well) and are likely heritable because the characteristics were present in both patients and relatives (cf. 131). However, endophenotype research in SAD is new; nevertheless, given the results of family and twin studies showing genetic influences in the development of SAD ^132– 135^, such work is of importance in order to gain insight into the genetic susceptibility to SAD.

In the Leiden Family Lab study on Social Anxiety Disorder ^136^, we investigated candidate endophenotypes of SAD ^137^ by using a multiplex, multigenerational family design (MRI sample: eight families, n = 110). Within the sample, we explored evidence for two endophenotype criteria: the co-segregation of the endophenotypes with social anxiety, within families genetically enriched for SAD, and the heritability of the endophenotypes. This study revealed multiple promising neurobiological endophenotypes of SAD ^138^. To start, several structural brain characteristics, derived from cortical and subcortical brain regions, co-segregated with social anxiety within families and were at least moderately heritable ^139^; see accompanying commentary ^140^. Furthermore, we employed two fMRI paradigms to explore the potential of brain responses as SAD endophenotypes. Using the first paradigm, the Social Norm Processing Task (revised version ^141^; based on work by 142, 143), we found evidence for hyperreactivity of the medial PFC and frontal pole in response to unintentional social norm violations as a neurobiological endophenotype of social anxiety ^144^. Second, we investigated responses to neutral faces. Data revealed that impaired neural habituation in the hippocampus met the two endophenotype criteria of interest ^145^; in addition, amygdala engagement in response to conditioned faces with a social-evaluative meaning qualified as a neurobiological candidate endophenotype of social anxiety ^146, 147^. Although future studies are required to examine the stability of these candidate endophenotypes over time (endophenotype criterion 2) and to discover genetic variants underlying the abovementioned candidate endophenotypes, these promising findings offer a starting point for follow-up studies on the genetic susceptibility to SAD.

## Outstanding questions and future research

The studies summarized above suggest that multiple brain regions are functionally or anatomically altered in patients with SAD (biomarkers) or involved in the genetic vulnerability to develop the disorder (endophenotypes). However, several important considerations remain and most of them are not specific for research on SAD but apply to the broader field of neuroimaging research in psychiatry ^21^. First of all, as recently outlined by Etkin in his thought-provoking review ^21^, meta-analyses of brain structure and function across psychiatry have shown that brain alterations are often non-specific ^148, 149^; that is, similar brain changes are apparent in distinct psychiatric disorders (based on DSM criteria); interestingly, recent studies implicate that the same non-specificity is present in the field of psychiatry genomics ^150, 151^. Therefore, it needs to be investigated whether a dimensional approach as described in the Research Domain Criteria (RDoC) framework ^152^, which focuses on symptom levels, could yield more reliable neurobiological biomarkers of specific clinical presentations.

A second important question concerns the issue of causality. Although studies with larger sample sizes could lead to more reliable results with respect to brain alterations related to psychopathology, their findings still concern associations and do not necessarily imply causal mechanisms ^21, 153^. For a more elaborate illustration of the risk of “just-so” stories (being “internally consistent explanations that have no basis in fact”), we recommend a recent viewpoint article ^153^. To increase our understanding of the functional implications of brain alterations in psychopathology, future research should combine neuroimaging with neurostimulation tools, which are able to intervene with normal brain functioning. Such tools (for example, non-invasive brain stimulation) enable causal relationships to be discerned and could reveal target points for interventions ^154– 156^.

This issue strongly relates to the final open questions that we wish to highlight, namely whether the SAD-related brain characteristics described above can be influenced in order to prevent the development of SAD or to alleviate its symptoms. It would be interesting to investigate whether a cutting-edge technique such as real-time fMRI-based neurofeedback ^157– 160^ could be successfully used in the prevention and treatment of SAD. In addition, a focus on individual level biomarkers, using new “precision MRI” approaches, offers promising prospects for optimizing diagnosis and treatment ^161– 163^. It is our hope that the insights from neuroimaging research will eventually lead to promising effective interventions that increase the quality of life of patients with SAD.

## Abbreviations

ACC, anterior cingulate cortex; BNST, bed nucleus of stria terminalis; CBT, cognitive behavioral therapy; DSM, *Diagnostic and Statistical Manual of Mental Disorders*; ENIGMA, Enhancing Neuro Imaging by Meta-Analysis; FC, functional connectivity; fMRI, functional magnetic resonance imaging; MRI, magnetic resonance imaging; PFC, prefrontal cortex; SAD, social anxiety disorder
